# Predominance of *Brucella abortus* antibodies among women with spontaneous abortion in the city of Mwanza: unrecognized link or coincidence?

**DOI:** 10.1186/s13104-018-3906-4

**Published:** 2018-11-06

**Authors:** Fridolin Mujuni, Venance Andrew, Elifuraha B. Mngumi, Elieza Chibwe, Stephen E. Mshana, Mariam M. Mirambo

**Affiliations:** 1 Department of Obstetrics and Gynecology, Weill Bugando School of Medicine, P.O. Box 1464, Mwanza, Tanzania; 2 Department of Microbiology and Immunology, Weill Bugando School of Medicine, P.O. Box 1464, Mwanza, Tanzania; 3 Department of Veterinary Pathology, Sokoine University of Agriculture, P.O. Box 3018, Morogoro, Tanzania

**Keywords:** Brucella, Antibodies, Spontaneous abortion, Mwanza, Tanzania

## Abstract

**Objective:**

This study investigated the association of Brucella seropositivity and spontaneous abortions in human population in the city of Mwanza, Tanzania.

**Results:**

A comparative cross sectional study which used 148 sera from women with spontaneous abortion and 250 sera from full-term delivered women was conducted in July 2017. Detection of *Brucella abortus* and *Brucella melitensis* antibodies was done using slide agglutination test. Data were analyzed using STATA version 13 software. The median age of the study participants was 25 (interquartile range 21–30) years. The overall seropositivity of Brucella antibodies was significantly higher among sera from women with spontaneous abortion than full term delivered women; (86/148, 58.1%: 95% CI 50–66 vs. 65/250, 26%: 95% CI 18–33, P < 0.001). Seropositivity of *B. abortus* was significantly higher among sera from women with spontaneous abortion than full-term delivered women (31.8% vs. 10.8%, P < 0.001). Women with abortion had 3.59 odds of being brucella seropositive compared to full term women (OR: 3.59, 95% CI; 2.25–5.74, P < 0.001). Seropositivity of Brucella antibodies is significantly higher among women with spontaneous abortion than full-term delivered women necessitating a need to investigate the relationship between Brucellosis and adverse pregnancy outcomes.

## Introduction

Brucellosis is a neglected bacterial zoonotic disease that is common in the Sub Saharan African countries [1, 2]. Brucellosis is of public health concern particularly in the agropastoral communities. It is associated with economic losses and reduced productivity as a result of spontaneous abortions in animals such as goats, sheep and cattle [3]. The disease is widespread in Western Europe, sub-Saharan Africa, Middle East, Central Asia and South America [2, 4–7]. The incidence of human brucellosis in endemic regions ranges from < 0.01 to > 200 per 100,000 population [8]. In Tanzania, the prevalence of brucellosis in human population has been found to be as high as 46% among livestock keepers [9].

In humans, the clinical presentation of the disease varies from asymptomatic to severe life threating disease [1, 10]. Brucellosis is well established cause of spontaneous abortion in animals particularly ruminants, however, its role in causing spontaneous abortions in human is unclear [11]. Abortion in animals is mainly due to the presence of high content of erythritol in animal placental tissues which is believed to play an important role in the localization of *Brucella* spp. [12]. In human, it is believed that maternal bacteremia, toxin production, disseminated intravascular coagulation and acute febrile reaction contribute to spontaneous abortion and intrauterine fetal death [13–15].

Bone marrow cultures has been considered as the gold standard for the diagnosis of brucellosis [16], nevertheless, harvesting bone marrow for culture is an invasive procedure and unreliable. The serum agglutination test remains to be widely used diagnostic method whereby the titers above 1:160 are considered diagnostic parallel with clinical presentations [17].

Spontaneous abortion is a condition which occurs in about 20% of women before 20 weeks of pregnancy with the majority of them occurring in the first 12 weeks of gestation [18]. The rate of spontaneous abortion in Tanzania is estimated to be approximately 12%, however the causes of these abortions have never been thoroughly studied [19]. Infectious agents such as *Toxoplasma gondii*, viral infections including rubella, cytomegalovirus, parvovirus B19 and many other agents have been implicated as common causes of spontaneous abortions [20–23]. Brucellosis is considered to be endemic in the sub-Saharan region including Tanzania and it has been associated with other adverse pregnancy outcomes such as stillbirth [24, 25]. However, its role in spontaneous abortion has never been established. This study for the first time in Mwanza attempted to compare seroprevalence of brucella antibodies between women with spontaneous abortion and that of full term delivered women. This information might be useful as a baseline information and may stimulate further studies to establish the pathogenesis and a clear link between brucellosis and abortion in human.

## Main text

This was a comparative cross-sectional study, carried out in July 2017 in the city of Mwanza, Tanzania. A total of 148 and 250 archived sera from women with spontaneous abortion and full-term delivery women, respectively were retrieved and analyzed for brucella antibodies (*B. abortus* and *B. melitensis*). Samples were collected from four health facilities in the city of Mwanza namely Bugando Medical Centre, Sekou Toure regional hospital, Buzuruga health centre and Nyamagana hospital between 2015 and 2016.

Sample size was estimated using Kish Leslie formula using prevalence of 20% from a previous study in Kenya [26]. The minimum sample size obtained was 245, however a total of 398 sera were available and included in the study. Sera with complete information including storage conditions were serially collected and analyzed. Socio-demographic data and other relevant information were obtained from preexisting records.

Detection of brucella antibodies was done using slide agglutination test (SAT) as per manufacturer’s instructions (Euromedi equip LTD UK). Reagents and test samples were brought at room temperature for 20 min. Eurocell antigen suspensions was shaken and mixed well before dispensing. The Eurocell-A and Eurocell-M reagents contain ready for use standardized, attenuated Brucella antigen with specific reactivity towards antibodies to *B. abortus* (Eurocell-A), and *B. melitensis* (Eurocell-M). The test has 95% sensitivity and specificity of 100%.

Data were extracted from existing database in excel sheet, cleaned and coded then transferred into STATA version 13 for analysis. Percentage/fraction was used to summarize categorical variables while median (IQR) was used for continuous variables. Chi square test was used to show association between Brucella antibodies and associated factors followed by logistic regression analysis to establish adjusted odd ratio. All factors with P value less than 0.2 on univariate analysis were subjected on multivariate logistic regression analysis. A P value of < 0.05 at 95% confidence interval was considered as statistically significant.

The median age of the study participants was 25 (IQR 21–30) years. Regarding marital status, 126 (85%) and 215 (86%) of women with spontaneous abortion and full term delivered women were married, respectively. Regarding gravidity; the median number of pregnancies was 3 [IQR 1–4] for women with spontaneous abortion while for full term delivered women was 2 [IQR 1–3] pregnancies. Regarding HIV status, 39/85 (54.1%) and 39/85 (45.9%) had unknown HIV status among women with spontaneous abortion and full term delivered women, respectively (Table 1).

**Table 1:** Sociodemographic characteristics of the study participants

Characteristics	Abortion		Full term	
	Number/frequency	Percentage (%)/median (IQR)	Number/frequency	Percentage (%)/median (IQR)
Pregnancy status	148	37.19%	250	62.81%
Age	148	26.4 [25.4–27.3] years	250	25.2 [24.5–26.0] years
Gravidity	148	3 [1–4]	250	2 [1–3]
Marital status				
Married	126	85.1%	215	86%
Single	22	14.9%	35	14%
Education				
Primary	82	32.3%	172	67.7%
Secondary	48	44.0%	61	56.0%
Tertiary	18	51.4%	17	48.6%
Occupation				
Business	27	18.24%	88	35.2%
Employed	23	15.54%	21	8.4%
House wife	61	41.22%	74	29.6%
Peasant	37	25.0%	67	26.8%
HIV status				
Positive	1	7.7%	12	92.7%
Negative	101	33.7%	199	66.3%
Unknown	46	54.1%	39	45.9%

The overall seropositivity of Brucella specific antibodies among sera from women with spontaneous abortion was 86/148 (58.1%, 95% CI 50–66) while that of full term delivered women was found to be 65/250 (26%, 95% CI 18–33), P < 0.001. Seropositivity of *B. abortus* was significantly higher among sera from women with spontaneous abortion than from full term delivered women [47/148 (31.8%) vs. (10.8%), P < 0.001]. Further analysis showed that there is no significant difference in seropositivity of *B. melitensis* among sera from women with spontaneous abortion and those from full term delivered women [19/250 (7.6%) vs. 10/148 (6.8%), P = 0.381]. Mixed Brucella seropositivity (*B. abortus* and *B. melitensis*) was found to be significantly higher among sera from women with spontaneous abortion than full term delivered women [29/148 (19.5%) vs. 19/250 (7.6%), P = 0.001].

Regarding factors associated with Brucella seropositivity among the entire population studied, only type of population was significantly associated with seropositivity. It was found that women with abortion had 3.59 odds of being brucella seropositive compared to full term women (OR: 3.59, 95% CI; 2.25–5.74, P < 0.001) (Table 2).

**Table 2:** Factors associated with seropositivity of Brucella antibodies among 398 participants

	Univariate		P value	Multivariate		P value
	Positive	Negative		OR	95% CI	
Age	26 [22–30] years	24 [20–30] years	0.109	0.98	0.92–1.04	0.566
Gravidity	2 [1–4]	2 [1–3]	0.044	1.10	0.89–1.35	0.356
Education						
Primary	85 (33.5%)	169 (66.5%)		1		
Secondary	52 (47.7%)	57 (52.3%)		1.45	0.57–3.65	0.425
Tertiary	14 (40.0%)	21 (60.0%)	0.036	0.79	0.30–2.08	0.639
Occupation						
Peasants	37 (32.2%)	69 (66.4%)		1		
H/wife	56 (41.5%)	79 (58.5%)		0.68	0.28–1.60	0.374
Business	37 (32.2%)	78 (67.8)		0.74	0.31–1.77	0.503
Employed	23 (52.3%)	21 (47.7%)	0.072	0.66	0.28–1.56	0.349
M/status						
Married	136 (39.9%)	205 (60.1%)		1		
Single	15 (26.3%)	42 (73.7%)	0.051	2.13	0.99–4.57	0.05
HIV status						
Negative	115 (38.3%)	185 (61.7%)		1		
Unknown	35 (41.2%)	50 (58.8%)		0.94	0.54–1.62	0.818
Positive	1 (7.7%)	12 (92.3%)	0.066	0.15	0.02–1.27	0.083
Population						
Full term	65 (26.0%)	185 (74.0%)		1		
Abortion	86 (58.1%)	62 (41.9%)	< 0.001	3.59	2.25–5.74	< 0.001
History of abortion						
No	135 (38.1%)	219 (61.9%)				
Yes	16 (36.4%)	28 (63.6%)	0.819			

This is the first study in the city of Mwanza attempting to compare the Brucella seropositivity between women with spontaneous abortion and those with full term delivery. Overall seropositivity of brucella antibodies among sera from women with spontaneous abortion was found to be significantly higher than that of full term delivered women. The reported seropositivity from this study is higher than the previous reports in Iran, Nigeria and Kenya which documented the seropositivity of 13.6%, 19% and 27% among women with spontaneous abortion, respectively [14, 17, 27]. In addition, the reported seropositivity of *Brucella* spp. among women with normal deliveries in the current study was also found to be higher than what has been reported in Iran and Iraq which reported the prevalence of 6.2% and 15.2%, respectively [28, 29]. Variations in seropositivity in these studies can be explained by the differences in the diagnostic tests used [30]. The current study used Eurocell slide agglutination test with specificity of 100% and 95% sensitivity.

In the current study, seropositivity of *B. abortus* was significantly higher among sera from women with spontaneous abortion than that of sera from full term delivered women. This observation is different from a previous study in the United Kingdom [31]. Regarding *B. melitensis* there was no significant differences in seropositivity among the studied groups. The observed seropositivity is comparable to a previous report in the United Kingdom which reported seropositivity of 17.4% [31]. Seropositivity of mixed infection (*B. abortus* and *B. melitensis*) was found to be higher among sera from women with spontaneous abortion than among full term delivered women which is similar to a previous report [32]. In the current study on the multivariate analysis, we observed women with abortion to have 3.59 odds of being Brucella seropositive compared to full term women, however history of miscarriage was not found to be associated with brucella seropositivity. This could be explained by the timing of miscarriage and ability of the test to detect brucella antibodies from a distant past infection. Further studies to explore on the risk factors among these populations are highly recommended in Mwanza.

In conclusion the seropositivity of *B. abortus* antibodies is terrifyingly high among women with spontaneous abortion which call for the need for further investigations on the role of brucellosis in relation to adverse pregnancy outcomes particularly in resource limited countries where the disease is endemic.

## Limitations

Due to the design of the study correlation between the timing of miscarriage and seropositivity was not determined and many known factors of Brucellosis could not be assessed. In addition, detection of acute Brucella infection using molecular techniques was not done.
